# Producing Bilayer Graphene Oxide via Wedge Ion-Assisted Anodic Exfoliation: Implications for Energy and Electronics

**DOI:** 10.1021/acsanm.3c03284

**Published:** 2023-10-31

**Authors:** Daheng Zhang, Sankar Sasidharan, Jiahao Shi, Assa Aravindh Sasikala Devi, Jianhua Su, Jinhai Huang, Zhenyuan Xia

**Affiliations:** † Laboratory for Advanced Materials and Institute of Fine Chemicals, School of Chemistry & Molecular Engineering, East China University of Science & Technology, Shanghai 200237, P. R. China; ‡ Department of Industrial and Materials Science, Chalmers University of Technology, Göteborg 41296, Sweden; § Nano and Molecular Systems Research Unit (NANOMO), University of Oulu, 90014 Oulu, Finland; ∥ Shanghai Taoe Chemical Technology Co., Ltd., Shanghai 200030, P. R. China

**Keywords:** graphite intercalation compound, bilayer graphene oxide, cations, electrochemical oxidation, binding energy, energy storage

## Abstract

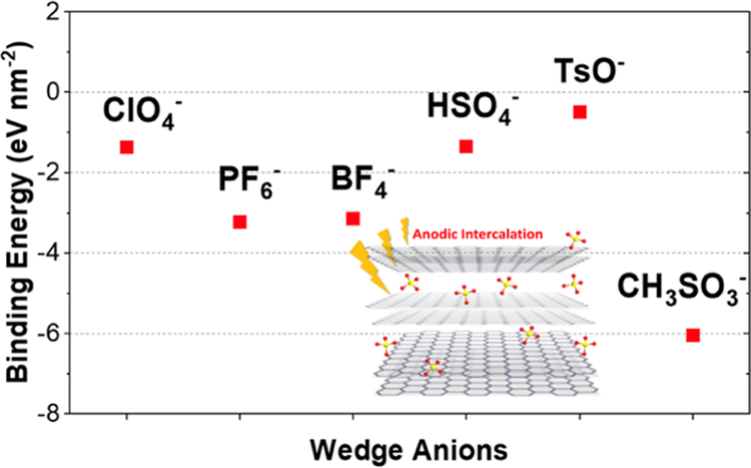

Electrochemical synthesis has emerged as a promising approach for the large-scale production of graphene-based two-dimensional (2D) materials. Electrochemical intercalation of ions and molecules between graphite layers plays a key role in the synthesis of graphene with controllable thickness. However, there is still a limited understanding regarding the impact of intercalant molecules. Herein, we investigated a series of anionic species (i.e., ClO_4_^–^, PF_6_^–^, BF_4_^–^, HSO_4_^–^, CH_3_SO_3_^–^, and TsO^–^) and examined their wedging process between the weakly bonded layered materials driven by electrochemistry. By combining cyclic voltammetry, X-ray diffraction (XRD), and Raman spectroscopy, along with density functional theory (DFT) calculations, we found that stage-2 graphite intercalation compounds (GICs) can be obtained through intercalation of ClO_4_^–^, PF_6_^–^, or BF_4_^–^ anions into the adjacent graphene bilayers. The anodic exfoliation step based on ClO_4_^–^–GIC in (NH_4_)_2_SO_4_ (aq.) resulted in the formation of bilayer-rich (>57%) electrochemically exfoliated graphene oxide (EGO), with a high yield (∼85 wt %). Further, the physicochemical properties of these EGO can be readily customized through electrochemical reduction and modification with different surfactants. This versatility allows for precise tailoring of EGO, making it feasible for energy and electronic applications such as electrodes in electrochemical capacitors and functional composites in wearable electronics.

## 1 Introduction

Recently, graphene and other two-dimensional (2D) materials, with their atomic thickness, have garnered considerable research and industry interest due to their exceptional and still non-fully explored properties. These outstanding physical and chemical properties such as high specific surface area, excellent mechanical and thermal stability, and electrical conductivity, make them ideal candidates for application in diverse fields such as energy storage and conversion, environmental remediation, and electronics.^1^ To exploit these layer-dependent properties, numerous techniques have been developed for the synthesis of graphene-related 2D materials in the last 20 years, where these methods are mainly attributed to two strategies, including bottom-up and top-down. Bottom-up methods involve building up the graphene structure by forming covalent bonds between individual atoms or molecules such as chemical vapor deposition (CVD) and epitaxial growth,^2,3^ while top-down methods could produce graphene directly from bulk graphite materials, achieved through techniques such as micromechanical exfoliation, liquid-phase exfoliation, chemical oxidation, and electrochemical exfoliation.^4−7^ Both strategies have advantages for different intended applications of graphene. For example, bottom-up methods are advantageous in applications that need precise control over the graphene structures at the atomic level with high quality, such as electronic devices.^8^ Alternatively, top-down methods are more suitable for large-scale production of solution-processable graphene with varying degrees of defects, making them beneficial for utilizing graphene as reinforcing additives in a wide range of composite applications.^9^

Among various top-down approaches, electrochemical synthesis is one of the most promising approaches for the large-scale production of 2D materials with tunable properties.^10,11^ Generally, it is an intercalation-assisted method for the fabrication of graphene-based layered 2D materials. During this process, guest molecules or ions can be intercalated into the graphite layers under the electric potential, weakening the van der Waals interactions between the interlayers, and facilitating further steps of graphene exfoliation.^6,12−14^ Depending on the electrochemical conditions, anodic and cathodic exfoliation has been successfully implemented. For anodic intercalation, some commonly used intercalant anions include sulfate (SO_4_^2–^), bisulfate (HSO_4_^–^), perchlorate (ClO_4_^–^), and tetrafluoroborate (BF_4_^–^), in either acid or salt form.^15−19^ Sulfate and bisulfate anions are the most widely used intercalants for scalable graphene exfoliation, particularly in aqueous solution of acidic (H_2_SO_4_) or neutral inorganic salts (i.e., (NH_4_)_2_SO_4_).^20−23^ Meanwhile, cationic intercalants such as alkylammonium (i.e., tetrabutylammonium, TBA^+^) and large metal ions (i.e., Cs^+^) in salt form are commonly employed ones for cathodic exfoliation in organic solvents.^24,25^ More recently, dual-electrode exfoliation via alternating applied potential or simultaneous anodic and cathodic intercalation on both graphite electrodes has been developed to improve the production efficiency.^26,27^ Besides, the co-intercalation of anionic complexes combining metal cations and chelating anions (i.e., [Mg(TFSI)_3_]^−^) also has shown their potential in anodic intercalation of graphite electrodes.^28^ The above molecules or metal ions act like “wedges” driven by the electric field and insert into the graphite layers through edges, causing an increased interlayer spacing. This intercalation process serves as a critical initial step in achieving a high yield of graphene. However, the specific role of these molecular wedges during the exfoliation process is still unclear. Moreover, electrochemical approach often yields graphene oxide (GO) with a higher thickness (1–2 nm) compared to that of the GO (≈0.9 nm, single layer) obtained by traditional chemical exfoliation like Hummers’ method.^16,17,21,24,25,29^ Thus, the feasibility of electrochemical exfoliation to primarily yield single-layer graphene sheets remains uncertain and requires further investigation.

Previously, we studied the impact of HSO_4_^–^ and SO_4_^–^ electrolytes in aqueous solutions during electrochemical exfoliation of graphite.^22,30^ Our findings revealed that the electrochemically exfoliated graphene oxide (EGO) sheets primarily consist of multiple structures of graphene bilayers. In this study, our focus is to investigate the initial intercalation step that occurs during the electrochemical exfoliation process in organic solvents consisting of a propylene carbonate (PC) and dimethyl carbonate (DMC) mixture. Specifically, we investigated the intercalation behavior of various anionic molecular wedges, including anions with a tetrahedral geometry: ClO_4_^–^, BF_4_^–^, HSO_4_^–^; anions with octahedron geometry: hexafluorophosphate (PF_6_^–^); and sulfonate-based anions with either methyl or bulky *p*-tolyl groups: methanesulfonate (CH_3_SO_3_^–^), *p*-toluenesulfonate (TsO^–^). The influence of these wedges on the intercalation stages and the stability of the corresponding graphite-intercalated compounds (GICs) were comprehensively studied by cyclic voltammetry, X-ray diffraction (XRD), Raman spectroscopy, and density functional theory calculations. By utilizing a mild electrochemical condition (+5 V for 1 h) and low concentration (0.1 M) of the intercalants, we can effectively form low-stage GIC complexes while minimizing the environmental impacts. Following the intercalation step, the stage-2 ClO_4_^–^–GIC complexes with moderate stability were chosen as the intermediate for the second step of electrochemical oxidation and exfoliation in aqueous solution. After the two-step anodic exfoliation process, the obtained EGO sheets are primary bilayer structures with good dispersibility in both *N,N*-dimethylformamide (DMF) and ethanol solvents. Finally, the EGO sheets could be easily functionalized with two types of organic surfactants and were subsequently treated by electrochemical reduction. This versatile electrochemical process has the potential to enhance the electrochemical behavior of EGO, making it an ideal choice as an electrode material in electrochemical supercapacitors or as an additive in polymer composites for flexible electronics.

## 2 Results and Discussion

### 2.1 Bilayer Graphene Intercalation Study Using Different Molecular Wedges

**Figure 1:**
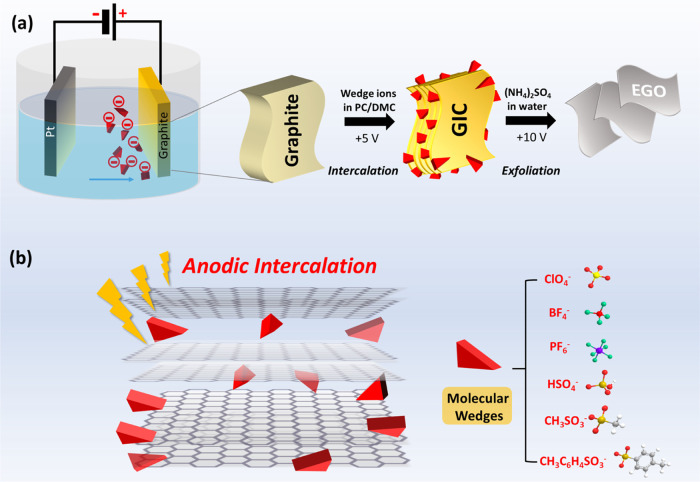
(a) Schematic illustration of the two-step intercalation and exfoliation anodic exfoliation process. (b) illustration of the anodic intercalation process with different molecular wedges.

**Figure 2:**
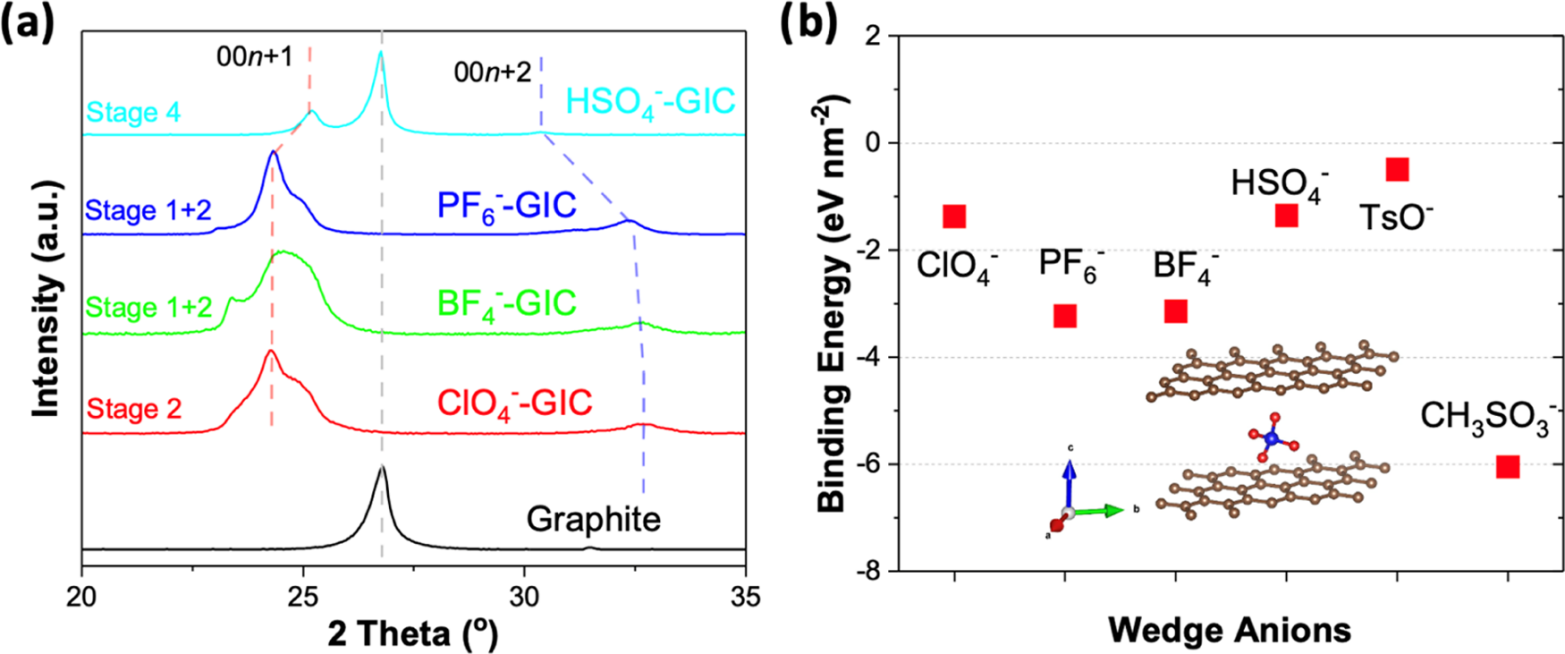
(a) X-ray diffraction patterns of the raw graphite and different GICs after electrochemical intercalation at 5 V for 1 h and (b) the corresponding binding energies of the six anion wedges; inset: calculated structure of the single-stage ClO4-based GIC.

To understand the intercalation behavior of various molecular wedges with bulk graphite through the anodic intercalation, a range of tetrabutylammonium (TBA) salts with different anion species, including ClO_4_^–^, PF_6_^–^, BF_4_^–^, HSO_4_^–^, CH_3_SO_3_^–^, and TsO^–^, were selected (Figure 1). A piece of graphite foil (1 cm × 1 cm, 0.5 cm thickness) was connected in a two-electrode cell with platinum foil (1 cm × 1 cm) as the counter electrode. The relative molecular salts (0.1 M) were dissolved in a mixture solvent of propylene carbonate (PC) and dimethyl carbonate (DMC) (1:3) as electrodes. After potentiostatic charging (1 h, 5 V), the GIC compounds were immediately evaluated by X-ray diffraction. As shown in Figure 2a, the dominant graphite (002) peak originally centered at 26.5° vanished after intercalation with ClO_4_^–^, PF_6_^–^, and BF_4_^–^ ions. Two new peaks were formed, one intensive peak (00*n* + 1) at lower Bragg angles and another less intensive (00*n* + 2) peak at higher angles positioned within the range of 24.2 and 33.4°. In the case of PF_6_^–^, and BF_4_^–^ intercalation, two extra peaks were observed at 23.4 and 35.2°. Meanwhile, HSO_4_^–^-intercalated GIC still maintained the original graphite (002) peak, with two extra peaks at 25.2 and 30.4°. These peaks clearly indicate the periodic repeat unit in graphite known as the staging transformation (Table S1). The stage number (*n*) of the GICs can be calculated according to the (00*n* + 1) and (00*n* + 2) peaks with the following formula:
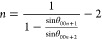
1Accordingly, the periodic unit distance *I*_c_ can be calculated with the gallery height (*d*_i_) of the intercalated graphene plane or the calculated interlayer distance (*d*_00*n*+1_)
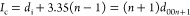
2The stage intercalation calculation demonstrates that ClO_4_^–^, PF_6_^–^, and BF_4_^–^ wedges predominantly result in the formation of stage-2 GIC, corresponding to the wedge ions in every two graphene interlayers. Additionally, a minor presence of stage 1 GIC is also confirmed in PF_6_^–^ and BF_4_^–^ wedges, indicating the existence of wedge ions in each graphene interlayer. However, HSO_4_^–^ only partially arrives at stage 4 GIC under the same electrochemical condition. And there is no intercalation behavior observed in CH_3_SO_3_^–^ and TsO^–^ wedges. This suggests that different wedges have a significant impact on the exfoliation of graphite.

To better understand the role of different anodic wedges during the intercalation process, we investigated the structures and energetics of these anodic intercalated GICs by density functional theory calculations in two different conditions: with and without a solvation effect.

The implicit solvation method as implemented in vaspsol is used for calculations.^31,32^ The binding energy (*E*_b_) of intercalants was calculated using the following equation (Table S2 and Figures S1–S6)
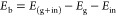
3where *E*_(g+in)_ represents the total energy of the supercell that contains both graphene and intercalating molecules, *E*_(g)_ indicates the total energy of the pristine graphite supercell, and *E*_(in)_ represents the total energy of the free-standing intercalant. Further, we have also performed implicit solvation-based calculations using VASP, and the binding energies are listed in Table S2. Figure 2b shows the optimized *E*_b_ of graphite with different wedges including the solvation effect, which exhibits a descending order as follows: CH_3_SO_3_^–^ > PF_6_^–^ > BF_4_^–^ > ClO_4_^–^ > HSO_4_^–^ > TsO^–^. A negative binding energy indicates that the intercalation is exothermic and energetically favorable, while a positive binding energy indicates an endothermic reaction and the intercalation requires extra energy to be given to the system. Most of the anions show similar binding energy results either with or without implicit solvation effect, except for CH_3_SO_3_^–^ (vacuum calculation: *E*_b_ = 0.82 eV for; solvent calculation: *E*_b_ = −6.52 eV) and ClO_4_^–^ (vacuum calculation: *E*_b_ = −11.21 eV for; solvent calculation: *E*_b_ = −1.37 eV). We believe that this is due to the introduction of the continuum dielectric that controls the averaging over molecular configurations. This approach, embedded in static solvation framework, helps mitigate the impact of the oxygen- or hydrogen-containing radicals in ClO_4_^–^- and CH_3_SO_3_^–^-based models. The results of our PF_6_^–^, BF_4_^–^, and ClO_4_^–^–GIC data involving solvation effect are consistent with the other recent reports involving COMSOL package and implicit solvation model.^27^

**Figure 3:**
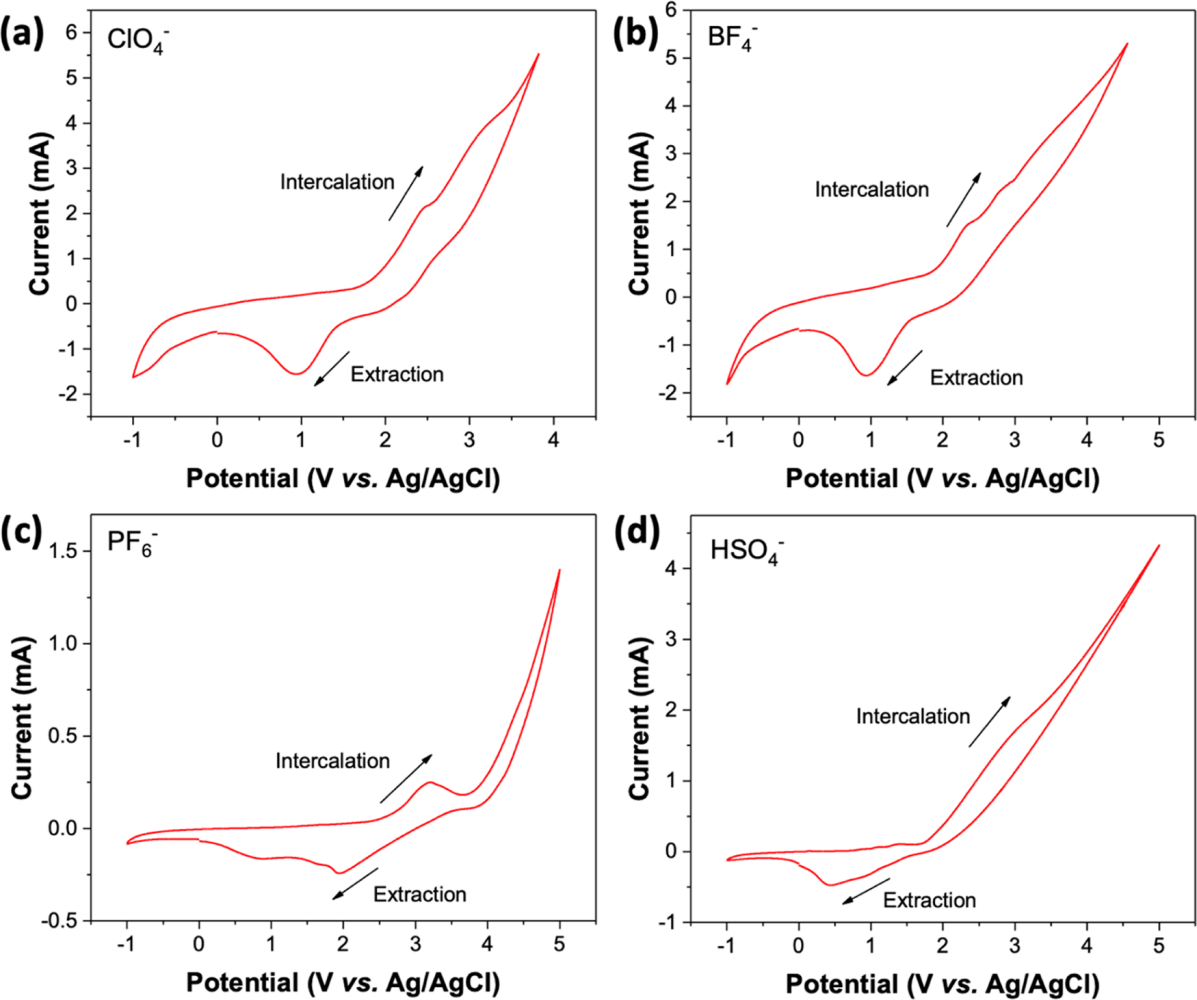
Cyclic voltammetry of the graphite electrode in (a) 0.1 M TBAClO_4_, (b) 0.1 M TBABF_4_, (c) 0.1 M TBAPF_6_, and (d) 0.1 M TBAHSO_4_ in PC-DMC solvent at the scan rate of 50 mV s^–1^ with Pt foil as counter electrode and Ag/AgCl as reference electrode.

The intercalation behavior of different molecular wedges was further studied by cyclic voltammetry (CV) with the scan range from −1 to 5 V using Ag/AgCl reference electrodes. Figure 3a–c clearly shows a set of redox peaks observed in ClO_4_^–^ (anodic peak: ca. 2.4 V; cathodic peak: ca. 1.0 V)-, BF_4_^–^ (anodic peak: ca. 2.3 V; cathodic peak: ca. 0.9 V)-, and PF_6_^–^ (anodic peak: ca. 3.2 V; cathodic peak: ca. 1.9 V)-based electrolytes during the voltammetric scanning. It is noticed that the anion intercalation voltage for PF_6_^–^ is ca. 4.5 V vs Li/Li^+^.^18,19^ Theoretically, we can convert Li/Li^+^ into the Ag/AgCl scale according to the difference of their standard electrode potential difference (−3.04 V for Li/Li^+^ and +0.23 V for Ag/AgCl). However, the Li/Li^+^ potential in nonaqueous solvents could be significantly affected by the solvent–electrolyte interaction, electrolyte salt, and lithium concentration.^33^ Consider that we used 0.1 M TBAPF_6_ in PC/DMC, which differs from the conditions in the two previously reported studies (e.g., 1 M LiPF_6_ in EMC). Therefore, we hypothesize that the variation in electrolyte conditions may account for the difference between our measured onset potential and the converted one from Li/Li^+^.

In the case of the bisulfate ions, a cathodic peak at ca. 0.4 V and a broad anodic peak at ca. 2.7 V were observed (Figure 3d). These anodic peaks are associated with the intercalation of the corresponding molecular anion wedges with the formation of GIC compounds. On the other hand, the cathodic peaks are attributed to the extraction of molecular wedges with the deformation of the respective GIC compounds. The intercalation/extraction behavior was also found on CH_3_SO_3_^–^ based sulfonic anions, with two broad anodic peaks (ca. 2.2 and 2.7 V) and one cathodic peak (ca. −0.6 V) (Figure S7a). However, there was no intercalation performance observed for the TsO^–^-ion-based wedges (Figure S7b). The above results suggest that (i) ClO_4_^–^ and BF_4_^–^ anion-based wedges have low onset intercalation potentials for the formation of GIC compounds, which means that the intercalation of these molecules needs less energy than the others to overcome the van der Waals interaction between the graphene interlayers; (ii) PF_6_^–^, HSO_4_^–^, and CH_3_SO_3_^–^ anions need more energy to achieve the intercalation of graphite, with the corresponding higher onset intercalation potentials; (iii) TsO^–^ anions cannot intercalate into the graphite layers due to its structural steric hindrance. Our previous calculation (Table S2) also reveals that the interlayer distance for TsO^–^-based GIC is 9.36 Å. Such a high distance requires higher intercalation energy for TsO^–^ to penetrate between graphite layers, which cannot be achieved under the current intercalation potential condition.

**Figure 4:**
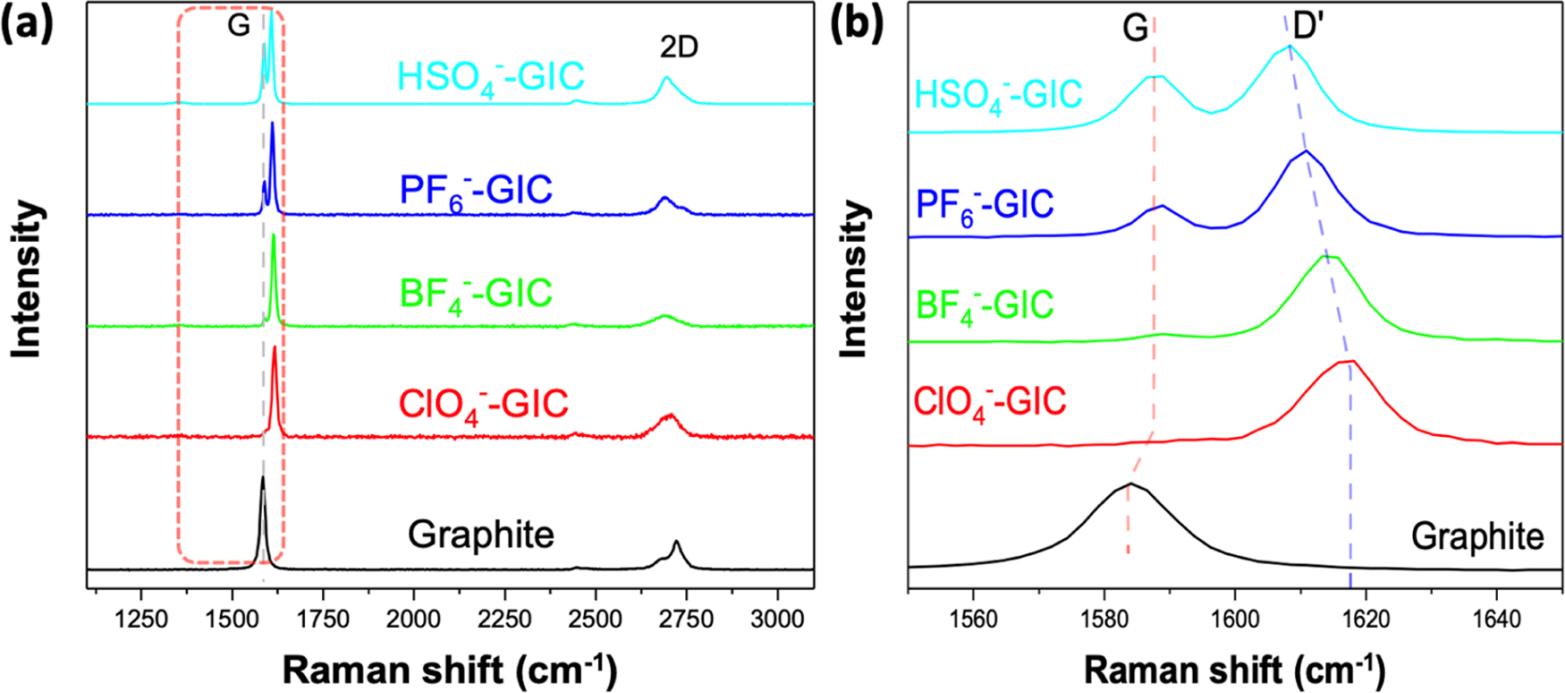
Raman spectra of different GICs by electrochemical intercalation at +5 V for 1 min: (a) whole spectra and (b) enlarged details of the G band range.

The intercalation process of different molecular wedges was also characterized by using Raman spectroscopy. Raman technique has emerged as a powerful tool for understanding the chemical composition and molecular structure of graphene and its derivatives. As shown in Figure 4a, after electrochemical intercalation at 5 V for 1 min with different electrolytes, Raman spectra of graphite surface exhibited 3 peaks: graphite peak (G), intercalation peak (D′), and 2D peak. The G peak at ca. 1583 cm^–1^ for raw graphite is a typical signature of in-plane vibration of the sp^2^ hybridized carbon structure. Following the intercalation of anion molecules, the G peak split into two peaks (G peak at ca. 1587 cm^–1^ and D′ peak at ca. 1608–1617 cm^–1^), which indicated the formation of low-stage GIC (Figure 4b). The ratio of the normalized intensities of G and D′ peaks for ClO_4_^–^, BF_4_^–^, PF_6_^–^, and HSO_4_^–^ anion intercalated graphite, *I*_G_/_D′_, is 0, 0.08, 0.29, and 0.62, respectively. According to the *I*_G_/*I*_D′_ ratio, the stage number *n* for the four anions above can be calculated to be 2.0, 2.16, 2.58, and 3.24. The intercalation result fits well with the previous XRD data that stage-2 intercalation could be easily achieved for ClO_4_^–^, BF_4_^–^, and PF_6_^–^ wedges, while higher-stage intercalation (stage 4) was monitored for HSO_4_^–^ wedges. The intercalation process for HSO_4_^–^ anion was also conducted for a longer time (5 min) to determine if a lower stage could be achieved (Figure S8). After 5 min of intercalation, a new D peak appeared, indicating the slight oxidation of the graphite surface during the HSO_4_^–^ intercalation process. Simultaneously, the relative peak ratio between the graphite peak (G) and the intercalation peak (D′) changed from 0.62 to 1.1. These results suggest that longer oxidation times in organic electrolytes (PC/DMC) do not lead to lower intercalation stages for HSO_4_^–^, but the oxidation of the uppermost graphite layers and the continuous intercalation of the inner layers. This observed phenomenon agrees with our previous study on the anodic intercalation of graphite in H_2_SO_4_ and Li_2_SO_4_ solution.^30^ Meanwhile, the blue shift of the D′ peaks with the decrease of stage number is due to an increasingly positive electronic charge density of the graphene layer. As for CH_3_SO_3_^–^ and TsO^–^ anions, there was no observation of G peak changes after intercalation at the same condition. It is worth mentioning that the visibility of the D peak (also known as the defect peak, at ca. 1330 cm^–1^) is negligible in all of the intercalated samples within 1 min, indicating almost no oxidation or damage of the graphite crystalline structure during the intercalation process in short period.

### 2.2 Electrochemical Production of Bilayer Graphene Oxide after Intercalation

The formation of a low-stage (*n* ≤ 2) GIC is crucial for achieving a high-yield exfoliation of graphite. This is because it enables the pre-expansion of graphite interlayers when the selected molecular wedges occupy the graphitic galleries along the *c*-axis direction, and the larger interlayer spacing facilitates the diffusion of other intercalation agents in the further electrochemical exfoliation process. However, direct graphite intercalation and exfoliation in aqueous electrolytes (i.e., HSO_4_^–^/SO_4_^2–^ in acidic media and SO_4_^2–^ in neutral media) are unable to achieve low-stage GIC due to the inhibiting effect of water. It results in the intercalation process being halted until a stage *n* ≥ 5 is reached, leading to a low yield production of graphene sheets.^30^ Therefore, it is necessary to separate the intercalation and exfoliation process into two steps, in which organic electrolytes are used for the GIC formation, while aqueous electrolytes are used for the expansion and exfoliation of graphene sheets.

During the first step, anodic wedges penetrate the inside of the graphite structure through the graphite grains or step edges, in which these anions are ultimately arranged parallel to the neighboring bilayer graphene. The use of organic electrolytes offers a notable advantage in the intercalation process by providing a wide electrochemical stability window, which means that no competitive side reactions caused by water electrolysis will occur. In the second step, other wedges like HSO_4_^–^ or SO_4_^2–^ replace the existing anions in the GIC matrix. The expanded graphite gallery height from GIC complexes facilitated subsequent intercalation with other molecules. Meanwhile, electrolysis of the co-intercalated water molecules accompanied by the oxidative cleavage of graphite under high potential results in the rapid expansion and exfoliation of the bulk graphite sample.^6^

Among the above wedge anions, BF_4_^–^ and PF_6_^–^, ClO_4_^–^ anions could achieve a low-stage intercalation number (*n* ≤ 2). However, perchlorate-containing compounds are known to have the potential for explosiveness in their anhydrous state. In our case, the presence of organic solvents during the formation of GIC creates a “wet” environment, which in turn contributes to the stability of our intercalated graphite.^34^ Our solvation calculation also indicated that when compared to BF_4_^–^ and PF_6_^–^, ClO_4_^–^ ions with lower binding energy or higher repulsive binding energy can be extracted more easily between the graphene layers. In contrast, the fluorine-containing wedges, BF_4_^–^ and PF_6_^–^, also presented low-stage intercalation (*n* ≤ 2) during the GIC formation step. However, due to their higher binding energy, it is difficult to “squeeze out” BF_4_^–^ and PF_6_^–^ wedge ions with other anions like SO_4_^2–^ in an aqueous solution during the second step. We observed that the BF_4_^–^ or PF_6_^–^ intercalated graphite tended to detach easily from the working electrode in the second step of the electrochemical exfoliation process, making it difficult to be fully exfoliated. Thus, ClO_4_^–^ was chosen as the intercalation agent of graphite in organic electrolytes, followed by the subsequent exfoliation step in aqueous electrolytes (0.1 M (NH_4_)_2_SO_4_). In the second step, water serves as an important component in the production of electrochemically exfoliated graphene oxide (EGO). From our previous study, we know that water molecules not only act as the source of oxygen gas through water electrolysis to boost the physical expansion of graphite interlayers but also function as an attacking nucleophile during the oxidative hydrolysis of the GIC complex. After fully oxidative expansion and exfoliation of the graphite foil at 10 V (ca. 20–30 min), EGO sheets were collected and purified by washing with water and finally dispersed in either DMF or ethanol solvents with the assistance of mild sonication. A high yield (∼85%) of bilayer-rich EGO sheets was obtained after this two-step intercalation and exfoliation process.

**Figure 5:**
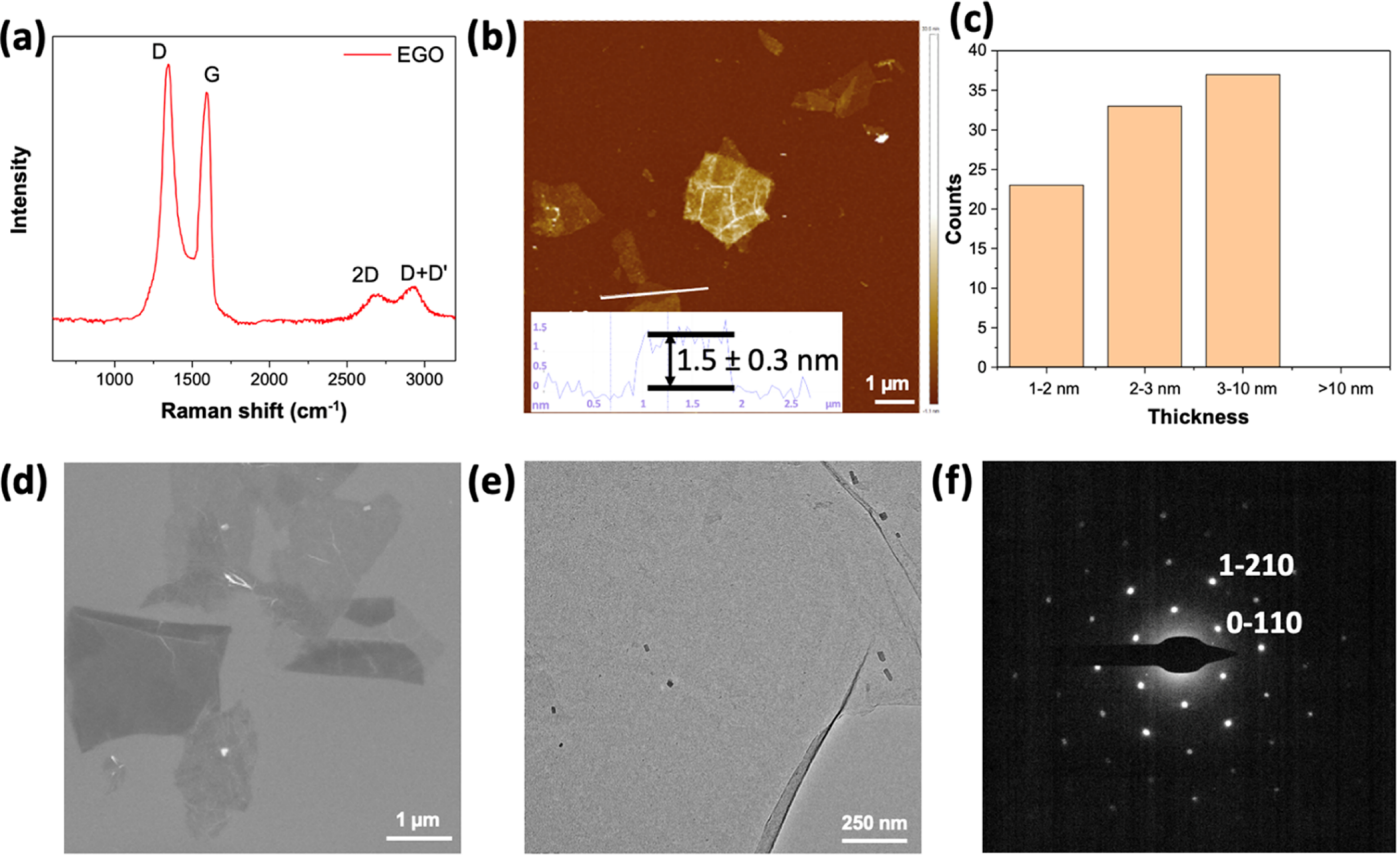
(a) Raman spectra of EGO after +5 V intercalation and +10 V exfoliation in the two-step exfoliation method; (b) AFM image of EGO sheets; (c) statistical thickness analysis of EGO based on 93 flakes; (d) SEM and (e) TEM images of EGO sheets; (f) SAED pattern from EGO.

To investigate the impact of applied intercalation potentials in the first step, we conducted experiments using a high applied potential at +10 V in addition to the intercalation at +5 V for the formation of ClO_4_^–^–GIC followed by the same exfoliation step. Raman analysis was used to identify the quality of obtained EGO sheets. As depicted in Figures 5a and S9, both the 5 and 10 V intercalation processes exhibit a prominent D peak and a slightly weaker G peak, along with clear 2D and D + G peaks. It is worth noting that reducing the applied potential during the first step in graphene foil leads to a decrease in the intensity of the D peak relative to the G peak. The intensity ratio of the D and G peaks (*I*_D_/*I*_G_) can be used to evaluate the graphene defects. In comparison to the 5 V intercalation (*I*_D_/*I*_G_ = 1.38), the 10 V intercalation yields a higher intensity ratio (*I*_D_/*I*_G_ = 1.76), indicating a greater degree of oxidation with more defects. This is likely attributed to the electrochemical oxidation that occurred during the intercalation step under a high potential.

The morphology of the prepared EGO after two-step intercalation (+5 V with ClO_4_^–^ in PC/DMC) and exfoliation (+10 V with SO_4_^–^ in water) was investigated by using scanning electron microscopy (SEM), atomic force microscopy (AFM), and transmission electron microscopy (TEM). For SEM and AFM analyses, EGO was dispersed in DMF and then spin-coated onto SiO_*x*_ wafer substrates. Figure 5b shows a representative AFM image of EGO, with an average thickness of ca. 2–3 nm and a lateral size of 1–2 μm. We also observed the thinnest flakes of around 1.5 ± 0.3 nm thickness. That value is in the middle of a GO bilayer (ca. 1.8 nm), which might come from the partially oxidized bilayer EGO sheets. We then examined the thickness of 93 flakes from the AFM images and categorized them according to their height. Figure 5c shows the statistical thickness analysis of EGO, that the majority of the flakes (57%) were thinner than 3 nm, corresponding to the multiple structures of bilayer EGO. However, some EGO flakes were found to be restacked together, resulting in higher thickness within the range of 3–10 nm. Similar flake size distributions were obtained from SEM images, as shown in Figure 5d. The above observation agrees with our previous findings that most EGO flakes are not single-layered. Furthermore, TEM analysis was used to identify the interlayer distance of the few-layer EGO flakes. Figure 5e displays a translucent EGO sheet with a crinkled appearance suspended on a TEM grid substrate. The layer spacing of the few-layer EGO is approximately 3.9 Å, as shown in Figure S10, which is slightly higher than the typical value for pristine graphite (3.4 Å). The result is consistent with our previous study on EGO samples, which could be explained by the partial oxidation of the defective graphene sheets.^22^ A selected area electron diffraction (SAED) of the EGO sheet in Figure 5f exhibits a typical 6-fold symmetric diffraction pattern, with more intense diffraction observed from the (1–210) plane than from the (0–110) plane, supporting our hypothesis that the EGO sheet possesses a bilayer crystalline structure.

**Figure 6:**
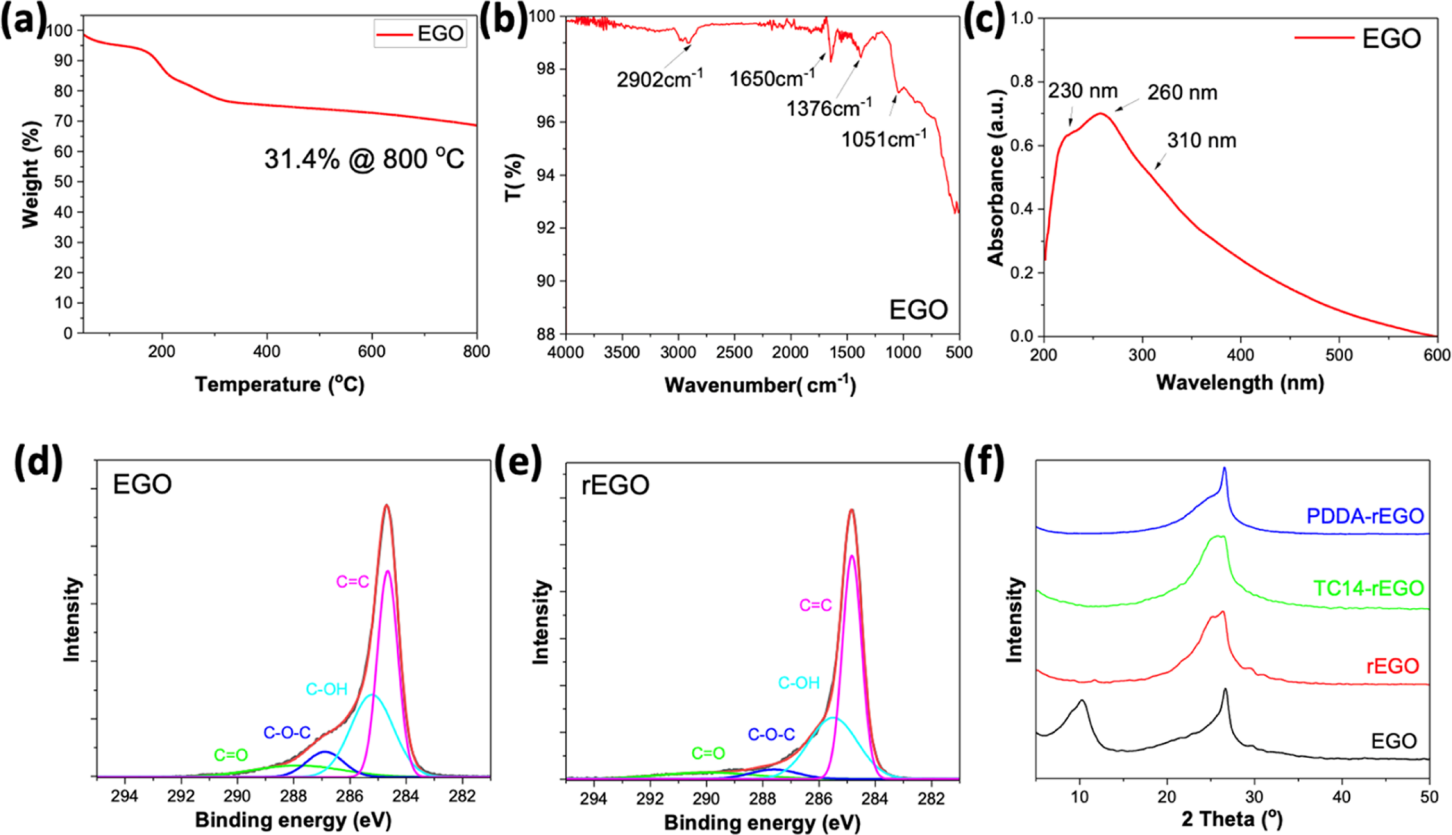
(a) TGA curve of EGO; (b) FTIR spectrum of EGO; (c) UV–vis spectrum of EGO; XPS C 1s spectra of (d) EGO and (e) rEGO; (f) XRD pattern of EGO, rEGO, TC14-rEGO, and PDDA-rEGO.

The thermal stability of EGO was investigated using thermal gravimetric analysis (TGA) to understand the temperature threshold at which oxygen-containing functional groups decompose. Figure 6a illustrates the significant weight loss of EGO starting from approximately 150 °C, with a total weight loss of 31.4% from 50 to 800 °C. The weight loss value is lower compared to values reported in the literature for electrochemically produced graphene oxide (34.6%) and chemically produced graphene oxide (42.2%).^21^ This difference can be attributed to the lower density of oxygen-containing functional groups on the surface of the EGO produced using the method described in this study.

The structural and optical properties of the EGO sheets were characterized by FTIR and UV–vis measurements. In the FTIR spectra of the EGO sample, as shown in Figure 6b, characteristic peaks are evident, corresponding to various oxygen-functional groups. These include the O–H stretching vibration of carboxyl groups (2902 cm^–1^), C=C vibration and skeleton vibration of benzene (1650 cm^–1^), C–H vibration (1376 cm^–1^), and C–O vibration (1051 cm^–1^). UV–vis spectra also serve as useful qualitative analysis tools for exfoliated graphene sheets in solvents. Typically, UV–vis spectra of graphene oxide (GO) samples exhibit two absorption peaks resulting from the specific electronic transitions, corresponding to the n–π* transition from epoxide groups (ca. 290–310 nm) and the π–π* transition from the sp^2^ carbon conjugation system (ca. 233 nm).^35^ In the case of EGO suspension in ethanol (0.05 mg/mL), we observed the absorption peak centered at ca. 260 nm, which is due to the π–π* transition of a larger sp^2^ conjugation than the normal GO sample (Figure 6c).^36,37^ Additionally, one subtle peak at 230 nm and one broad peak at about 310 nm were detected. This phenomenon can be attributed to the preservation of the carbon-rich conjugated system inherited from pristine graphene, leading to a lower degree of oxidation in EGO compared to GO.

### 2.3 Electrochemical Reduction and Modification of EGO for Electrochemical Capacitors

EGO can be considered as a moderately oxidized form of graphene compared to traditional graphene oxide in which the graphite lattice is fully oxidized by chemical oxidation. However, EGO still processes certain oxygen-containing functional groups on its surface, which limits its application in electrochemical energy storage devices that require high electrical conductivity. Therefore, it is necessary to reduce the obtained EGO to improve its conductivity. Herein, we investigate an electrochemical reduction method for the reduction of the EGO film in aqueous solution. The reduction process was conducted in a two-electrode system at −2 V for 30 min using a dilute H_2_SO_4_ solution (0.1 M), in which EGO film prepared by vacuum filtration (approximately 7 mg) was used as negative electrode, while a platinum foil served as the counter electrode. The reduced EGO (rEGO), together with EGO, was analyzed by X-ray photoelectron spectroscopy (XPS) to study the chemical composition changes after reduction. As shown in Figures S11 and S12, the oxidation degree, as determined by the oxygen content, decreased from 16.5 atom % for EGO to 12.1 atom % for rEGO. Consequently, the carbon/oxygen (C/O) ratio of rEGO increased from 4.8 to 6.9 after the reduction process, indicating the elimination of oxygen-containing functional groups. It is noticed that the C/O value is still lower than that of other reports from electrochemically exfoliated graphene (12.3) but higher than the value of GO (≈2). The deconvoluted XPS spectra of the C 1s spectra of EGO and rEGO (Figure 6d,e) reveal prominent peaks corresponding to C=C (284.6 eV), weaker peaks for C–OH (285.5 eV) and the O–C–O (286.8 eV), and a weak peak for the C=O (290 eV) functional group. After reduction, the peak intensity of the C–O–C group in rEGO (4.4 atom %) is lower than that of EGO (6.8 atom %). Meanwhile, the presence of a π to π* component at 291 eV in the C 1s spectrum indicates the retention of conjugated structures in both EGO and rEGO.^38^

XRD measurement further confirmed the successful reduction of EGO into rEGO. The EGO film prepared using the two-step electrochemical method displayed two distinct peaks at 10.2 and 26.6° (Figure 6f). The formal peak corresponds to the oxidized graphene with sp^3^ hybridization domains, while the latter peak, characterized by a broad shape, suggests the presence of sp^2^ graphitic domains in the EGO sample.^39^ The result agrees with our previous study that the moderate electrochemical oxidation process could create a partially oxidized graphene bilayer structure, which is composed of repeated bilayer partially oxidized graphene stacking with small fragments.^22^ For the rEGO sample, the diffraction peak at 10.2° vanished, and a broadened peak at 26.3° became dominant, indicating the efficient restoration of graphitic domains through the removal of oxygen-functional groups.

**Figure 7:**
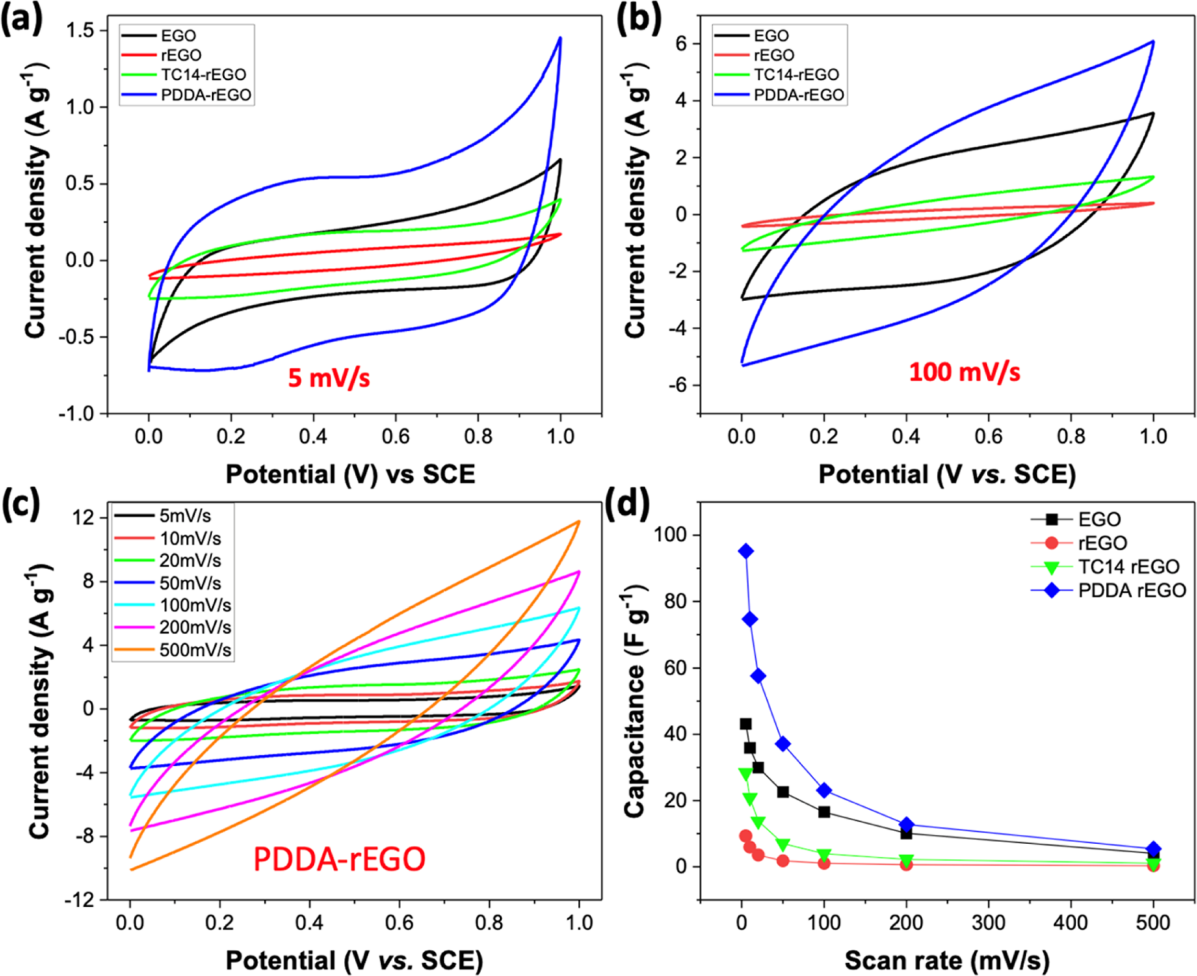
Electrochemical characterization of EGO, rEGO, rEGO, TC14-rEGO, and PDDA-rEGO film: CV curves of various samples at the scan rate of (a) 5 mV/s and (b) 100 mV/s; (c) CV curves of PDDA-rEGO at different scan rates; (d) specific capacitance vs scan rate curves for various samples.

The reduction of EGO was expected to enhance the electrical conductivity and thereby improve its performance for energy storage device applications. Thus, we evaluated the capacitance behavior of the EGO and rEGO film through electrochemical measurements in a three-electrode system using a 1 M Na_2_SO_4_ electrolyte. Both EGO and rEGO show typical electrical double-layer behavior with quasi-rectangular shapes through cyclic voltammetry (CV) scans at all sweeping rates from 5 to 500 mV/s (Figures 7a,b and S13). However, the specific capacitance of the rEGO film is much lower than that of the EGO film. The EGO has a specific capacitance of 43.1 F g^–1^ at a sweeping rate of 5 mV s^–1^, whereas the rEGO sample demonstrates a much lower specific capacitance of only 9.3 F g^–1^ at the same rate (Figure 7d). As is known, an electrical double-layer capacitor (EDLC) stores electrical energy by physically adsorbing ions onto the surface of the electrode. A higher surface area and larger interlayer distance in the EDLC could facilitate the diffusion and adsorption of the Na^+^ ion between the graphene layers. Our rEGO film might experience restacking of EGO sheets during the electrochemical reduction process. The π–π stacking of rEGO sheets results in less contact area between the electrode and water electrolytes and then impacts the overall capacitive behavior.

To avoid the face-to-face stacking of adjacent graphene sheets, two types of surfactants, one anionic surfactant 1,4-bis(neopentyloxy)-3-(neopentylcarbonyl)-1,4-dioxobutane-2-sulfonate (TC14) and one cationic surfactant poly(diallyldimethylammonium chloride) (PDDA), were added in the second step of electrochemical exfoliation with a concentration of 0.1 M to stabilize the as-prepared EGO separately. The obtained rEGO films with different surfactants after the filtration and reduction treatment were named TC14-rEGO and PDDA-rEGO, respectively. TC14 has been proved as an ideal surfactant for stabilizing graphene oxide, thanks to its unique triple-chain branched structure that could effectively prevent the aggregation of graphene flakes during the reduction process,^40^ while PDDA has been widely used as a linker for the self-assembly of 2D nanomaterials.^41^ XRD spectra of these films (Figure 6f) suggest that the TC14-rEGO diffraction peak has no apparent difference compared to the rEGO peak without TC14. A broadening of the XRD peak at 26.3° could be attributed to the anchoring of certain TC14 molecules inside the rEGO layers. As for the PDDA additive, the diffraction peak at 26.6° displays a profile that is quite similar to the EGO peak at the same position. However, a broad shoulder peak was observed at 24° in the PDDA-rEGO film, suggesting a more disordered layer structure with slightly expanded graphite interlayers.

The following CV scans of TC14-rEGO and PDDA-rEGO present that both additives improved the capacitance performance in comparison to pure rEGO. The specific capacitances of TC14-rEGO and PDDA-rEGO are 28.4 and 95.1 F g^–1^ at 5 mV s^–1^ and 3.9 and 23.1 F g^–1^ at 100 mV s^–1^, respectively (Figures 7a,b,d, and S13). Surprisingly, the addition of PDDA was found to enhance the capacitance of rEGO significantly, while the addition of TC14 did not show the same effect. To understand the influence of TC14, the XRD spectrum of the TC14-EGO film before reduction was recorded in comparison to that of TC14-rEGO. Besides the two identical peaks at 10.2 and 26.6°, which correspond to EGO, several sharp peaks (18.5, 21.7, and 25.2°) were detected superimposed on a board peak ranging from 15 to 24° (S14). These additional peaks could be attributed to the periodic anchoring of TC14 molecules within the graphitic structure of EGO. Based on these findings, it is hypothesized that the anionic TC14 molecules were partially extracted from EGO during the electrochemical reduction process and migrated toward the positive electrodes, leading to the partial aggregation of rEGO and the observed lower capacitance performance. In contrast, PDDA molecules were effectively incorporated into the negatively charged EGO sheets during the reduction due to their cationic properties. The electrostatic attraction between EGO and PDDA prevented the restacking of EGO itself and facilitated a larger contact area between the electrode and electrolyte. Moreover, the hydrophilic nature of PDDA is advantageous in promoting the transmission of ions and electrons within the water electrolytes. As a result, the PDDA-rEGO film shows the best electrochemical performance with high capacitance values (Figure 7c,d).

## 3 Conclusions

In general, the anodic intercalation of graphite with a range of anion species was performed to study the formation of low-stage GIC complexes. Stage-2 GIC with ClO_4_^–^ anions was easily achieved during the electrochemical intercalation process using organic electrolytes. The calculated low binding energy of ClO_4_^–^–GIC demonstrates the facile diffusion of perchlorate ions between graphene layers, thereby facilitating the intercalation of sulfate ions in the subsequent step. Further electrochemical oxidation and exfoliation process in aqueous solution yielded bilayer-rich (57%) electrochemically exfoliated graphene oxide (EGO) at a higher yield (>85%) and a moderate oxygen content (16.5 atom %). Our findings propose that a combination of efficient and nondestructive intercalation with subsequent oxidative expansion and exfoliation in different media (nonaqueous/aqueous electrolytes) offers a highly efficient approach for graphite exfoliation. Also, the EGO product can undergo cathodic reduction and additional functionalization by incorporating specific surfactants like TC14 and PDDA. This electrochemical modification capability enables the customization of EGO with various small molecules and polymers, thereby ushering new possibilities for its application in diverse areas, including the direct utilization of EGO film for EDLC capacitors and the integration of EGO with polymers to create conductive elastomers, catering to the needs of flexible and stretchable electronics.

## 4 Experimental Section

### 4.1 Materials

Graphite foil (0.5 mm, purity of 99.8%) was purchased from Goodfellow and is produced by compression of expanded graphite. Sodium perchlorate (AR, Alfa Aesar, NaClO_4_), tetrabutylammonium perchlorate (AR, Sigma-Aldrich, TBAClO_4_), tetrabutylammonium tetrafluoroborate (AR, Sigma-Aldrich, TBABF_4_), tetrabutylammonium hexafluorophosphate (AR, Sigma-Aldrich, TBAPF_6_), tetrabutylammonium bisulfate (AR, Sigma-Aldrich, TBAHSO_4_), tetrabutylammonium methanesulfonate (AR, Sigma-Aldrich, TBACH_3_SO_3_), tetrabutylammonium p-toluenesulfonate (AR, Sigma-Aldrich, TBA-TsO), and poly(diallyldimethylammonium chloride) solution (20 wt % in H_2_O, Sigma-Aldrich, PDDA) were purchased from Sinopharm Chemical Reagent Co., Ltd. Dimethyl carbonate (ultradry, Sigma-Aldrich, DMC) and propylene carbonate (ultradry, Sigma-Aldrich, PC) were purchased from Shanghai Titan Technology Co., Ltd. Ammonium sulfate (AR, Bide, (NH_4_)_2_SO_4_) was purchased from Shanghai Bide Pharmaceutical Technology Co., Ltd. Platinum tablet (25 mm × 25 mm × 0.1 mm) and Teflon electrode clamp were purchased from Shanghai Yueci Electronic Technology Co., Ltd. DC power (RPS6005C-2) was purchased from Shenzhen Meiruike Electronic Technology Co., Ltd.

### 4.2 Synthesis of Electrochemically Exfoliated Graphene Oxide (EGO)

The synthesis of EGO involves two electrochemical steps using graphite foil as the starting material. Initially, the foil is cut into slices measuring 1 cm × 2 cm and subjected to electrochemical oxidation in a 0.1 M NaClO_4_ electrolyte (DMC/PC = 3:1) with a platinum cathode at a voltage of 5/10 V for approximately1 h. The mixture of two solvents can suppress the decomposition of a single compound during the electrochemical intercalation process.^42^ During this process, we observed a slight expansion of the GP and a blue coloration on its surface, which became more pronounced upon tearing the graphite apart, indicating complete intercalation. The second step involves the exfoliation of the graphite intercalation compounds (GICs) in a 0.1 M (NH4)_2_SO_4_ aqueous solution at a potential of 10 V, leading to the formation of dark gray GO. After filtration and drying, the yield of GO is approximately 85%. In the case of TC14- and PDDA-modified rEGO, TC14 or PDDA was added during the second step at a concentration of 0.1 M.

### 4.3 Synthesis of 1,4-Bis(neopentyloxy)-3-(neopentylcarbonyl)-1,4-dioxobutane-2-sulfonate (TC14)

#### 4.3.1 Production of TC14 Ester

Trans-Aconitic acid (5 g, 51 mmol) and neopentyl alcohol (3 equiv, 13 g, 153 mmol) were dissolved in toluene (100 mL), and p-toluene sulfonic acid (0.99 g, 5.75 mmol) added. The reaction mixture was heated to 110 °C for 12 h, and the water generated during the reaction was removed via a Dean and Stark apparatus. To remove any remaining impurities, the reaction mixture was washed multiple times with a saturated sodium bicarbonate (NaHCO_3_) solution. The organic phase was then dried using magnesium sulfate (MgSO_4_), and the solvent was evaporated to yield an off-white oil. Purification of the product was achieved through flash column chromatography using a silica gel column and eluting with a mixture of 10% ethyl acetate and petroleum ether. ^1^H NMR (600 MHz, Chloroform-*d*) δ 6.92 (s, 1H), 3.95 (s, 2H), 3.83 (s, 2H), 3.81 (s, 2H), 3.70 (s, 2H), 0.90 (d, *J* = 5.0 Hz, 18H), 0.84 (s, 9H).

#### 4.3.2 Production of TC14

TC14 ester (5 g, 13 mmol) was dissolved in ethanol (100 mL), and water was added up to saturation. Na_2_S_2_O_5_ (2.2 equiv, 5.47 g, 28.6 mmol) and Na_2_SO_3_ (1.8 equiv, 2.97 g, 23.4 mmol) were then added, and the mixture was allowed to heat under reflux for 6 h. The solvent was completely removed to give a white solid product which underwent crude purification via Soxhlet extraction using dry distilled AcOEt. Further purification was achieved by dissolving in the minimum amount of dry MeOH and centrifuging at 6000 rpm for 30 min. The supernatant solution was decanted from residual salts, and the solvent was removed to yield a white solid. ^1^H NMR (600 MHz, Chloroform-*d*) δ 4.47 (t, *J* = 6.3 Hz, 1H), 3.88–3.83 (m, 1H), 3.83–3.79 (m, 2H), 3.78–3.72 (m, 2H), 3.70 (d, *J* = 6.7 Hz, 2H), 3.66 (d, *J* = 10.1 Hz, 2H), 0.86 (d, *J* = 1.7 Hz, 9H), 0.85–0.83 (m, 18H) (Figure S15).

### 4.4 DFT Methodology

To corroborate with experimental findings, density functional theory calculations were performed using VASP package.^43,44^ The electron exchange and correlations were treated using generalized gradient approximation (GGA) and the pseudopotentials were employed in the PAW approximation.^45,46^ Energy and force tolerances of 1 × 10^–6^ and 1 × 10^–3^ eV/Å were employed for ionic optimization. To simulate the graphite layers, a supercell of dimensions *a* = *b* = 7.39 Å and *c* = 21.711 Å was used which contains 36 C atoms. A Monkhorst pack K grid with dimensions of 9 × 9 × 1 was used for the Brillouin zone integration. Further the molecules such as BF_4_^–^, ClO_4_^–^, HSO_4_^–^, PF_6_^–^, Ch_3_SO_3_^–^, and TsO^–^ were intercalated between two C layers and optimized. The implicit solvation method using the vaspsol package is carried out for binding energy calculations. This is a parametrized approach where solvent molecules are replaced with a continuum dielectric, and an averaging is carried out over molecular configurations embedded in solvent model parameters. VASP software is based on solving the Kohn–Sham equations self-consistently to achieve the electronic ground state, and the solvent effects are included by modifying the local potential of the Kohn–Sham Hamiltonian, as well as the equations of the total free energy and forces. Thus, to include the effect of the solvent medium, the generalized Poisson equation should be included in the self-consistent loop, as the valence charge density changes in each iteration. Therefore, in this implementation in VASP, the generalized Poisson equation is solved to obtain the electrostatic potential of the electronic charge density of both solute and solvent.^31^

### 4.5 Electrochemical Intercalation Measurement

Cyclic voltammetry (CV) of the graphite intercalation/deintercalation experiments was recorded at a scanning rate of 50 mV/s in 0.1 M TBA^+^ X^–^ electrolyte (X: BF_4_^–^, ClO_4_^–^, HSO_4_^–^, PF_6_^–^, Ch_3_SO_3_^–^, and TsO^–^) with PC/DMC (1:3) mixed solvent, respectively. The working electrode was graphite foil (1 cm × 1 cm, 0.5 cm thickness). The reference electrode was Ag/AgCl electrode, and the counter electrode was Pt foil (1 cm × 1 cm, 0.1 mm thickness).

### 4.6 Electrochemical Capacitor Measurement

Cyclic voltammetry (CV) was carried out in 1.0 mol L^–1^ Na_2_SO_4_ water solution with a CHI-660E electrochemical system. The working electrode was the graphene film. The reference electrode was Ag/AgCl electrode, and the counter electrode was Pt foil. Specific capacitance values were calculated from the CV curves using the following equation:
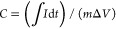
where *I* is the oxidation or reduction current, d*t* is time differential, *m* is the mass of the active graphene paper, and Δ*V* is the voltage range of one sweep segment.

### 4.7 Characterization

Scanning electron microscopy (SEM) images were obtained with a Helios G4 UC SEM-FIB instrument. Energy-dispersive X-ray spectroscopy (EDS) was carried out using 4 in-column Super-X detectors. Transmission electron microscopy (TEM) observations were carried out with a Thermo Fisher Talos F200X TEM (FETEM) at 200 kV. Atomic force microscope (AFM) characterization was performed using Bruker Dimension ICON. Raman scattering measurements were carried out with a laser micro-Raman spectrometer (model: inVia reflex ≤ *I*_0.2_/cm from Renishaw). X-ray photoelectron spectroscopy (XPS) was performed with an ESCALAB 250Xi from Thermo Fisher using a monochromatic Al Kα excitation source (XR6 specs). X-ray diffraction (XRD) was performed using Rigaku D/max2550VB/PC with copper target 18 KW (450 mA). Fourier Transform infrared spectroscopy (FTIR) was obtained from a Thermo Scientific Nicolet iS10 instrument with a spectra range from 7800 to 350 cm^–1^. Thermogravimetric analysis (TGA) was carried out using a PerkinElmer TGA8000 from room temperature to 800 °C. Cyclic voltammetry (CV) was performed using a CHI 760E electrochemical workstation, and ultraviolet–visible absorption spectroscopy was obtained with a SHIMADZU RF-6000.

## Supporting Information Available

DFT calculation data of GIC with different anions with and without solvation effect; XRD diffraction data; Raman spectrum; HRTEM micrographs, XPS survey spectrum, cyclic voltammetry profile, and H NMR data of TC14 (PDF)
