# Supplementary material for: Producing Bilayer Graphene Oxide via Wedge Ion-Assisted Anodic Exfoliation: Implications for Energy and Electronics
Source: ACS Appl Nano Mater. 2023 Oct 31;6(21):19639–50. doi: 10.1021/acsanm.3c03284 (PMC10644297; doi:10.1021/acsanm.3c03284)
Supplement: Supplementary file 1 — an3c03284_si_001.pdf [file an3c03284_si_001.pdf]

Supporting Information

Producing Bilayer Graphene Oxide via Wedge Ion assisted Anodic Exfoliation: Implications for Energy and Electronics

Daheng Zhang;[1] Sankar Sasidharan;[2] Jiahao Shi;[2] Assa Aravindh Sasikala Devi;[3] Jianhua Su;[1*] Jinhai Huang;[4*] Zhenyuan Xia[2*]

[1]*Key Laboratory for Advanced Materials and Institute of Fine Chemicals, School of Chemistry & Molecular Engineering, East China University of Science & Technology, Shanghai, 200237, PR China.*

[2]*Department of Industrial and Materials Science, Chalmers university of Technology, Göteborg, 41296, Sweden.*

[3]*Nano and Molecular Systems Research Unit (NANOMO), University of Oulu, 90014, Oulu, Finland.*

[4]*Shanghai Taoe Chemical Technology Co., Ltd, Shanghai, 200030, PR China.*

Table S1. XRD diffraction data of different GIC compounds with the calculated intercalant gallery height and stage numbers.

Intercalant	00*n*+1 (°)	00*n*+2 (°)	d_{00n+1} (Å)	Calc. Stage (n)	I_c (Å)	d_i (Å)
ClO_4^-	24.2	32.6	3.7	2	10.8	7.6
BF_4^-	24.5	32.6	3.6	2	11.2	7.6
	23.4	35.2	3.8	1	7.7	7.6
PF_6^-	24.3	32.4	3.7	2	11.2	7.6
	23.3	35.2	3.8	1	7.7	7.6
HSO_4^-	25.2	30.4	3.5	4	17.5	7.6
$CH_3SO_3^-$	-	-	-	-	-	-
TsO^-	-	-	-	-	-	-

Table S2. The calculated energies of GIC compounds with different molecular wedges

**Intercalant**	**Total energy $E_{(g+i)}$ (eV)**	**Energy of pristine graphite E_g (eV)**	**Energy of free-standing intercalant E_i (eV)**	**Distance between layers (Å)**	**Binding energy (eV)**	**Binding energy with solvation effect (eV)**	GIC unit
ClO_4^-	-352.85	-332.05	-9.59	7.10	-11.21	-1.37	$C_{36}ClO_4$
BF_4^-	-359.44	-332.05	-24.27	6.94	-3.12	-3.14	$C_{36}BF_4$
PF_6^-	-366.11	-332.05	-30.85	7.69	-3.21	-3.23	$C_{36}PF_6$
HSO_4^-	-365.51	-332.05	-32.44	7.50	-1.02	-1.35	$C_{36}PF_6$
$CH_3SO_3^-$	-373.53	-332.05	-42.30	9.27	0.82	-6.05	$C_{36}PF_6$
TsO^-	-443.55	-332.05	-110.78	9.36	-0.72	-0.49	$C_{36}PF_6$

Lattice parameters of supercell:
a= b= 7.392001052 Angstrom
c= 21.711000000 Angstrom
Volume = 1027.3879 Angstrom^3
Binding energy per (nm)^2 = - 0.05709927 eV /nm^2

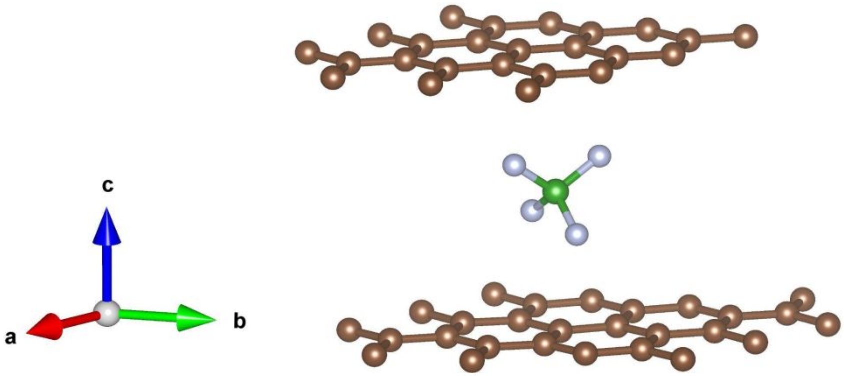

Figure S1. Relaxed structure of Graphite+BF_4, color codes: brown-C, green - B, silver- F

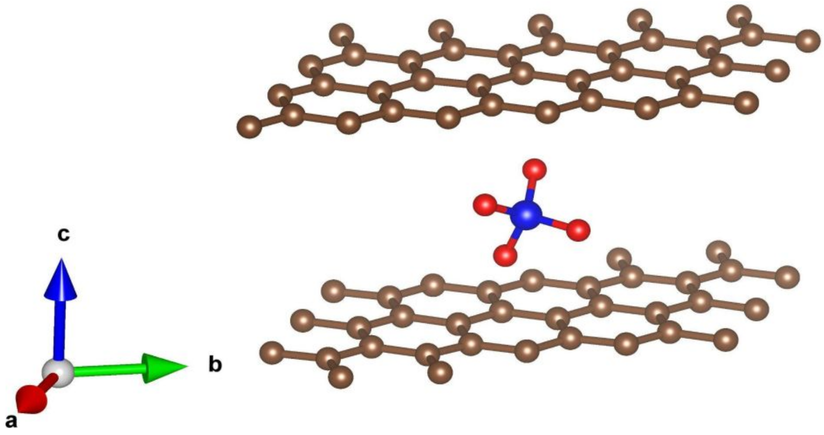

Figure S2. Relaxed structure of Graphite+ClO_4, color codes: brown- C, blue- Cl, red- O

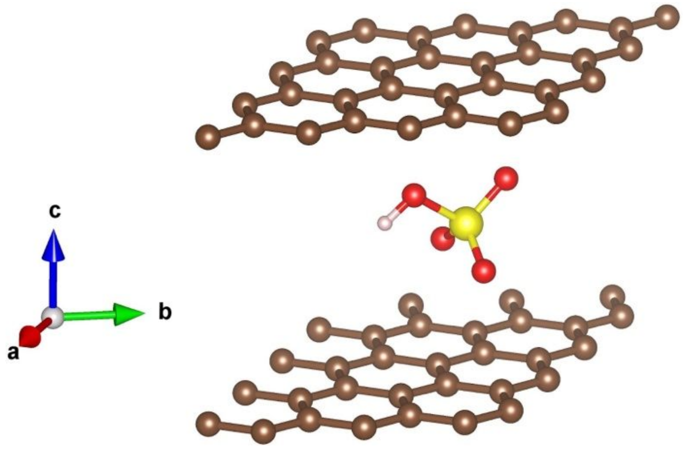

Figure S3. Relaxed structure of graphite+HSO_4, color codes: pink- H, yellow- S, red- O, brown- C

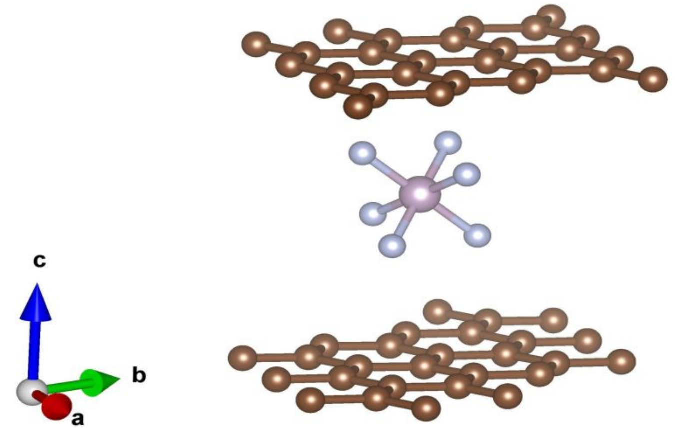

Figure S4. Relaxed structure of graphene +PF_6, color code: light pink- P, silver- F, brown- C

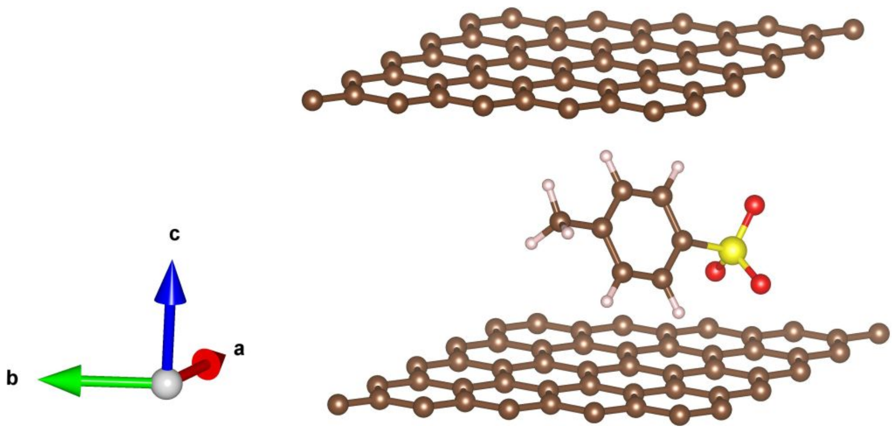

Figure S5. Relaxed structure of graphene + *p*-toluene sulfonic acid (TsO), color code: pink- H, yellow- S, red- O, brown- C

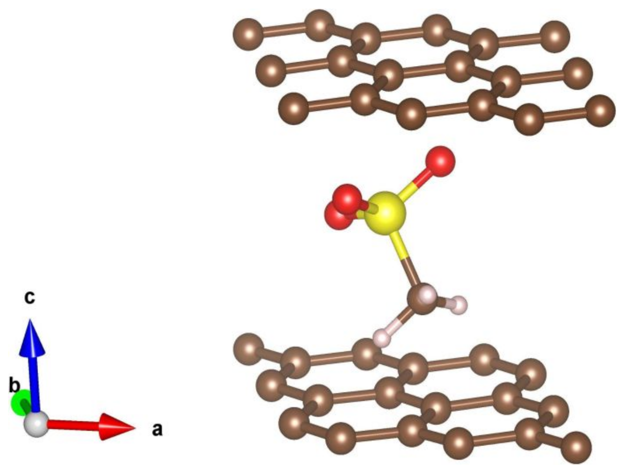

Figure S6. Relaxed structure of graphene + CH_3SO_3, color code: red- O, brown- C, yellow- S, pink- H

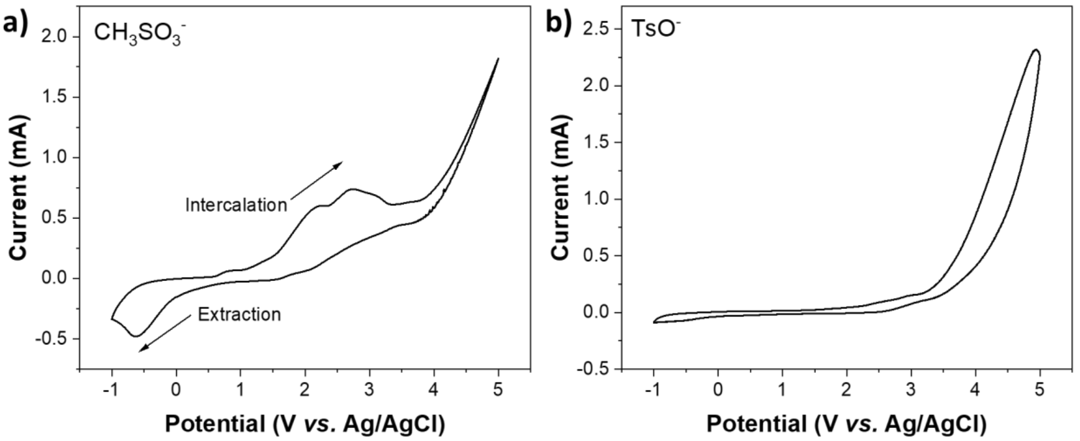

Figure S7. Cyclic voltammetry of the graphite electrode in (a) 0.1 M $TBACH_3SO_3$ (b) 0.1 M TBA-TsO in PC-DMC solvent at the scan rate of 50 mV s^{-1} with Pt foil as contour electrode and Ag/AgCl as reference electrodes.

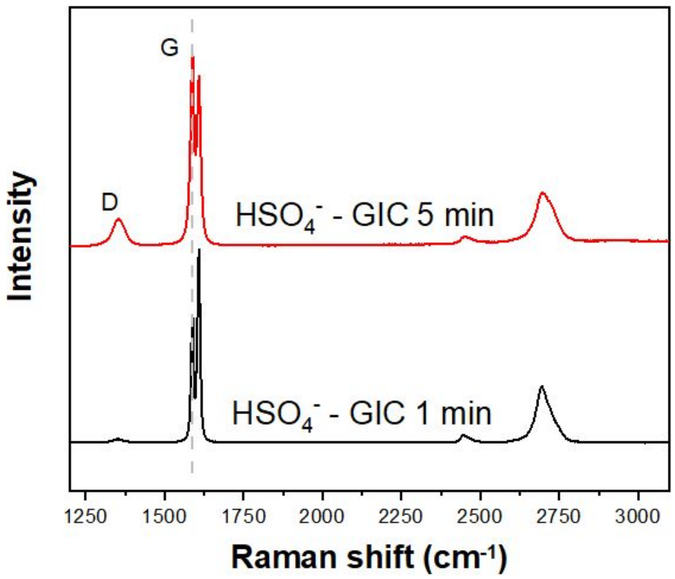

Figure S8, Raman spectra of graphite after anodic intercalation with HSO_4^- ion.

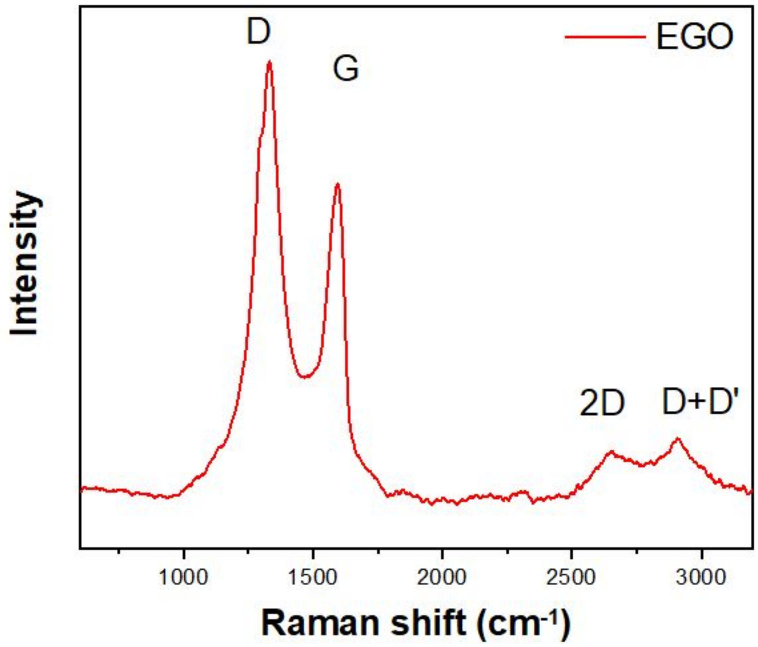

Figure S9. Raman spectra of EGO after +10 V intercalation and +10 V exfoliation in the two-step exfoliation method.

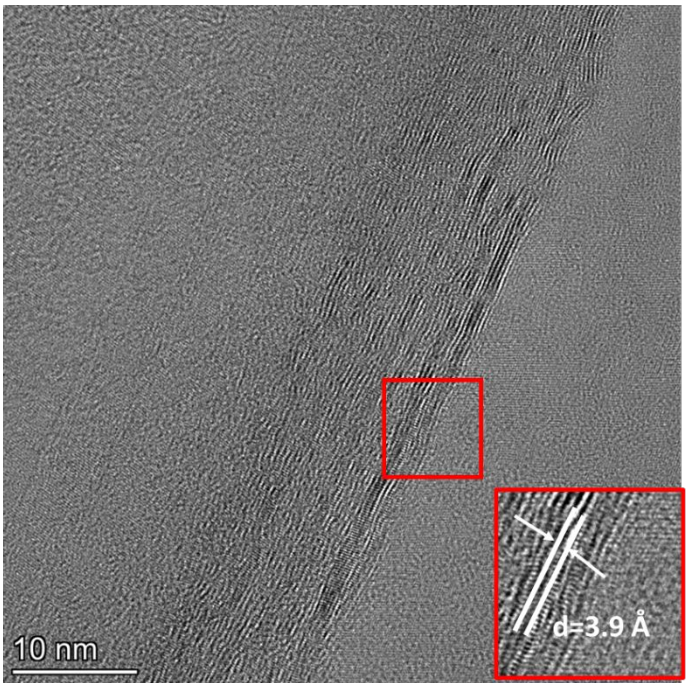

Figure S10. HRTEM image of few-layer EGO

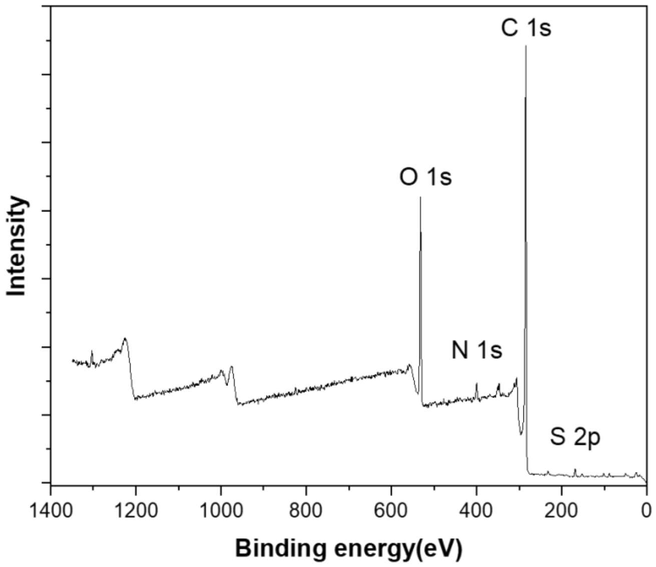

Element	Atomic %
C	79.0
O	16.5
S	3.5
N	1.0

Figure S11. Full survey XPS spectrum of EGO.

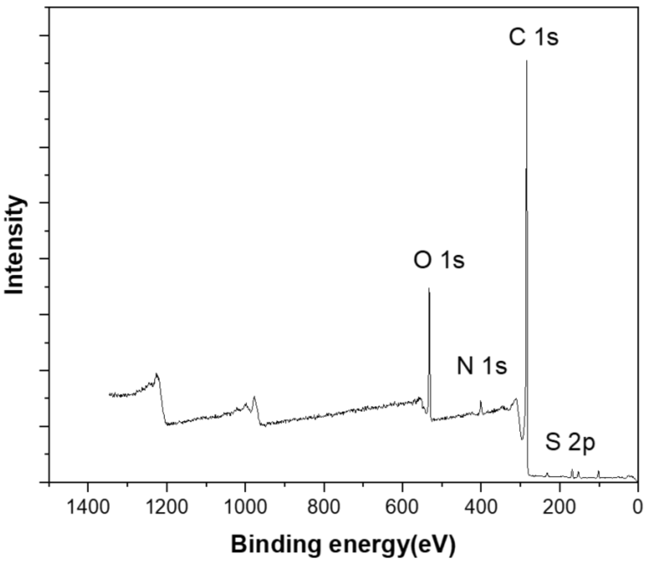

Element	Atomic %
C	83.8
O	12.1
S	3.1
N	1.0

Figure S12. Full survey XPS spectrum of rEGO.

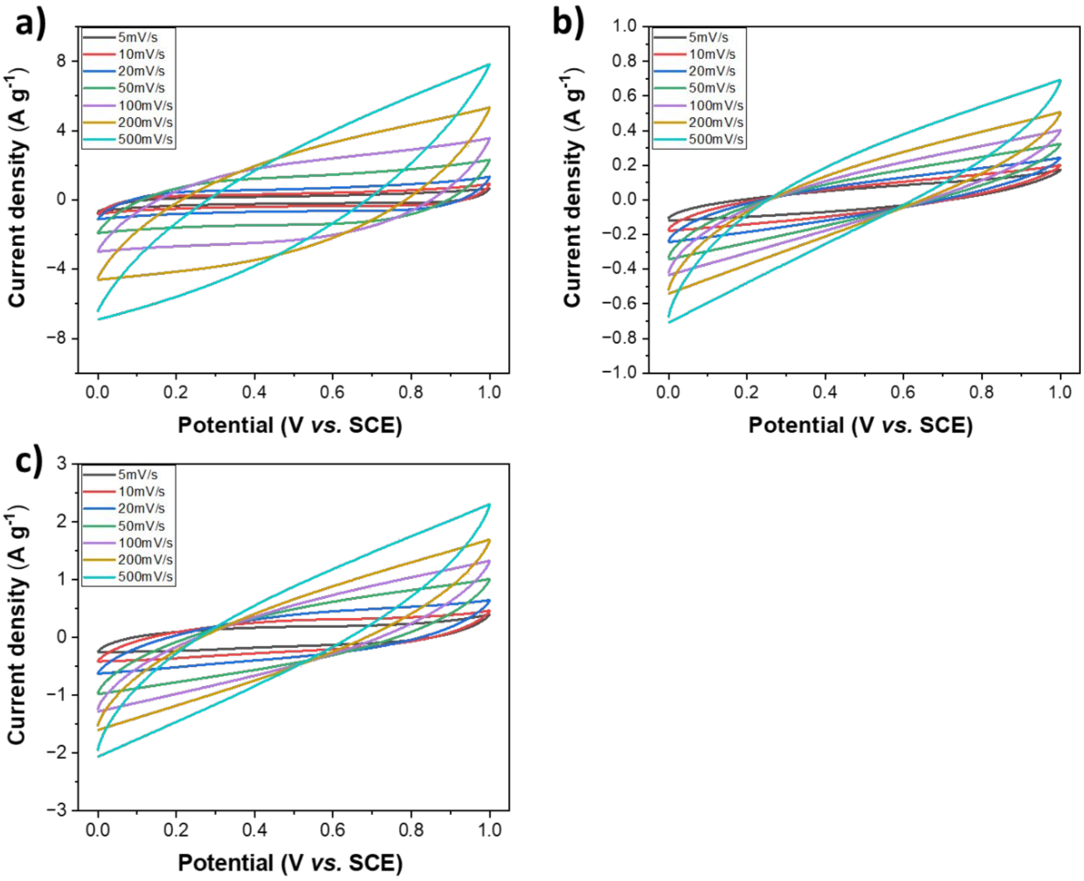

Figure S13. CV curves of a) EGO, b) rEGO, c) TC14-rEGO at different scan rates.

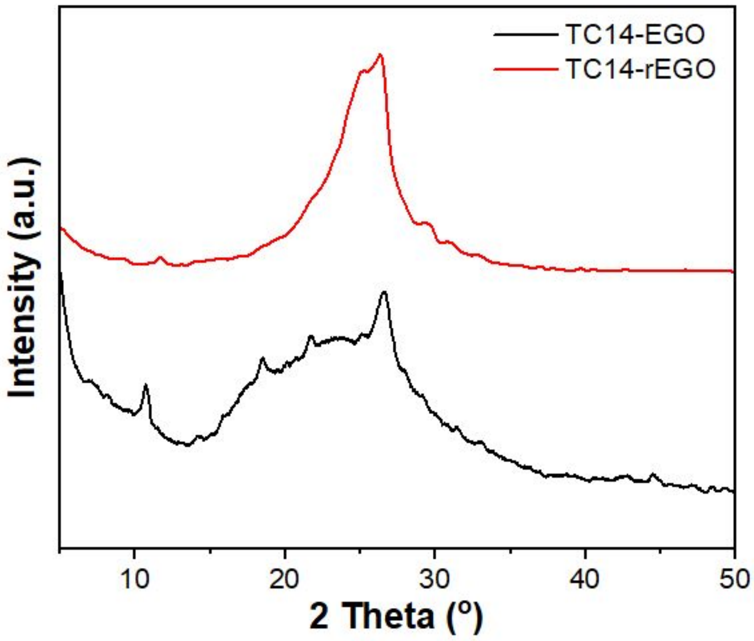

Figure S14. XRD pattern of TC14-EGO and TC14-rEGO.

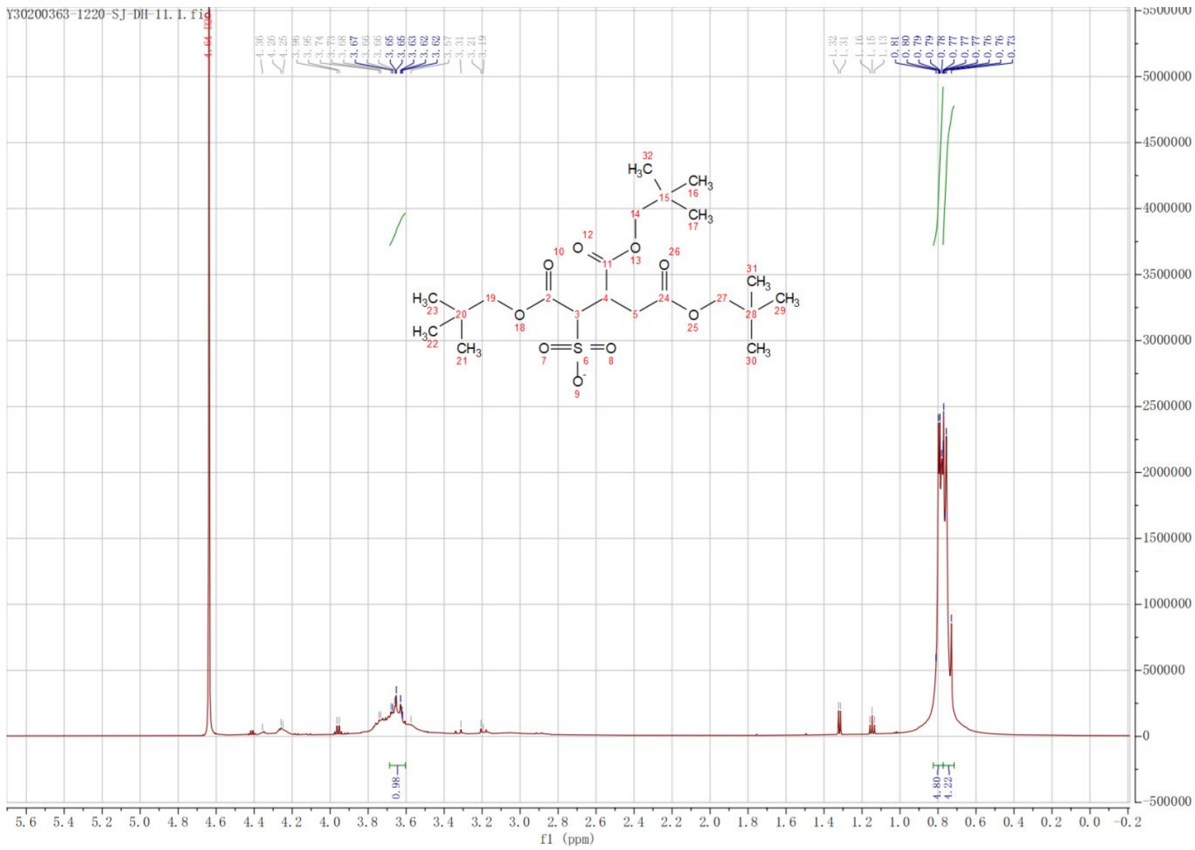

Figure S15. H NMR spectrum of TC14 in $CDCl_3$.
